# The association between energy-adjusted dietary inflammatory index and physical activity with sleep quality: a cross-sectional study

**DOI:** 10.1186/s40795-024-00834-0

**Published:** 2024-02-03

**Authors:** Mohammad Javad Zare¹, Seyed Jalil Masoumi, Morteza Zare

**Affiliations:** 1 Nutrition Research Center, School of Nutrition and Food Sciences, Shiraz University of Medical Science, Razi Boulevard, Shiraz, Iran; 2 Center for Cohort Study of SUMS Employees’ Health, Shiraz University of Medical Sciences, Shiraz, Iran; 3 Gastroenterohepatology Research Center, Shiraz University of Medical Sciences, Shiraz, Iran

**Keywords:** Diet, Dietary inflammatory index, Physical activity, Sleep, Sleep quality

## Abstract

**Background:**

The study aimed to assess the independent and interactive association of energy-adjusted dietary inflammatory index (E-DII) and physical activity (PA) with sleep quality.

**Method:**

A cross-sectional study was conducted on the 2466 participants (60% women). A 116-item food frequency questionnaire (FFQ) was applied to calculate E-DII, the International Physical Activity Questionnaire (IPAQ) long form for PA, and the Pittsburgh sleep quality index (PSQI) to assess sleep quality were collected via interview. Multivariate logistic regression was applied to assess independent and interactive associations of E-DII and PA with sleep quality.

**Result:**

No significant association was observed between E-DII and sleep quality (OR: 0.96, 95% CI: 0.92_1.01). Also, there was no significant association between the levels of PA and sleep quality. Women had 70% increased odds for poor sleep quality (OR: 1.7, 95% CI: 1.39_2.09) compared with men. No interactive association was observed between E-DII and PA levels with sleep quality.

**Conclusion:**

No significant association was observed between E-DII and PA levels with sleep quality. The study indicates a gender difference in sleep quality. Future prospective studies are required to confirm these findings.

## Background

Sleep is a physiological prosses with a critical role in brain function, endocrine, and immune systems [1]. Poor sleep quality is found to be associated with worse well-being and quality of life in addition to a wide range of diseases such as cardiovascular diseases (CVD), diabetes, and mental health [2–5]. Due to the importance of sleep various factors are under investigation for their potential role in sleep such as the role of environmental factors, physiological systems, and genetics [6–8].

Both nutrition and physical activity are (PA) among the potential influencing factors and their role in sleep is considerably under discussion. The impact of nutrition on sleep can be evaluated in different aspects such as the role of nutrient intakes like Vitamins and amino acids or adherence to different dietary patterns like the Mediterranean diet on sleep [9]. To investigate the association between dietary components and inflammation, the dietary inflammatory index (DII) has been developed to assess the inflammatory potential of diet [10]. Although several studies have been conducted to investigate the association between DII and sleep quality, the exact relationship is not clear due to the high contradiction among the results. Some studies reported a significant direct association between DII and sleep quality while others did not report any significant association [11–13]. Since elevated inflammatory markers such as C-reactive protein (CRP), interleukin-6 (IL-6), and tumor necrosis factor-α (TNF-α) was found to be associated with poor sleep quality [14–17], it hypothesized that higher adherence to a pro-inflammatory diet causes an increment in levels of CRP and IL-6 that may lead to poor sleep quality by inducing neuroinflammation [16–20].

In addition to nutrition, PA is one of the factors of interest in research on sleep quality. it’s suggested that PA can improve sleep quality potentially through its role in stress management [8, 21]. Moreover, higher PA levels can contribute to improved weight management, which in turn can lead to better sleep quality [22, 23]. Although there are numerous observational and experimental studies on the relationship between PA and sleep quality; associations differ when considering the participants’ age, health status, PA intensity, and assessment methods for both PA and sleep [24–28].

DII as an approach for dietary assessment and PA level as a measurement method for the estimation of PA are both indicators of nutrition and physical activity. There may be a possible overlap between a higher adherence to an anti-inflammatory diet and a higher physical activity level by improving systemic inflammation regulation and gut microbiota composition which can affect sleep [29–32]. So, understanding their independent and interactive impact on sleep quality may lead to the improvement of non-pharmacological interventions for sleep disorders [23, 33].

Although men and women are similar in many aspects of life, some biological and behavioral differences between them may impact health; for example, consideration of gender differences in the prevention and treatment of some diseases such as CVD and diabetes has been suggested previously [34–36]. Since higher poor sleep quality among women has been reported in some studies it was theorized that gender may play a role in sleep [37–39]. The exact association and underlying mechanism are not well understood but differences between sex hormones and their effect on circadian rhythms, stress, and inflammatory status can be considered as the assumptions for the possible relationship [40–43]. Therefore, we aimed to investigate the following hypotheses: (a) there is an independent and interactive association between E-DII and PA with sleep quality among participants and (b) there is a gender difference in sleep quality among participants.

## Methods

### Study population

Participants in the current study were derived from the Shiraz University of Medical Sciences Employee Health Cohort Study (SUMS EHCS) a branch of the PERSIAN Cohort Study as a secondary data analysis. The SUMS EHCS is an ongoing prospective cohort study started in 2017 in Shiraz, southern Iran. Participants were recruited by census method from SUMS employees aged between 20 and 60 years old. The study was conducted following the Declaration of Helsinki. All participants have provided written informed consent and the study was approved by the Ethics Committee of Shiraz University of Medical Sciences (Code: IR.SUMS.SCHEANUT.REC.1401.069).

For this cross-sectional study, we recruited data from 3293 participants of the SUMS EHCS enrolled in the first phase of the study from August 2018 to October 2019. Participants were excluded if they were night shift workers during the last month (*n* = 757), had incomplete or overestimation of physical activity for more than 16 h (*n* = 52), had incomplete or abnormal dietary intake (*n* = 16), and had incomplete data on sleep quality (*n* = 2). Finally, 2466 participants (975 male and 1491 female) were involved in the analysis.

### Dietary measurement

Dietary consumption was collected by a 116-item semi-quantitative food frequency questionnaire (FFQ) in a face-to-face interview with trained interviewers. The reliability and validity of this questionnaire have been confirmed in a previous study [44]. Participants were asked about each item’s frequency and amount of consumption daily, weekly, or monthly during the past year. An adapted version of Nutritionist IV diet analysis software (version 7.0; Nsquared computing, Salem, OR, USA) modified for Iranian food items was used to compute energy and nutrient intake.

### Energy-adjusted dietary inflammatory index (E-DII)

Shivappa et al. provided a quantitative measurement of diet using the association between inflammatory markers and 45 dietary factors based on 1943 articles. Each anti-inflammatory dietary parameter received a negative dietary score (-1) while pro-inflammatory parameters scored (+ 1) and if they had no effect scored as (0). More details are available elsewhere [10]. In this study, we calculated DII obtained from baseline FFQ data. Of the 45 possible dietary parameters, 29 parameters were available to calculate DII including; energy, carbohydrate, protein, total fat, fiber, cholesterol, saturated fat, monounsaturated fat, polyunsaturated fat, omega-3 fatty acids, omega-6 fatty acids, vitamin B12, vitamin B6, niacin, thiamin, riboflavin, iron, magnesium, zinc, selenium, vitamin C, vitamin E, vitamin D, folic acid, beta-carotene, caffeine, garlic, onion, and tea. We used participants’ dietary intake to calculate each participant’s intake of food parameters and compare it with the global reference database. To calculate the z-score for each participant, we subtracted the standard global mean from the dietary intake of each parameter and then divided it by the global standard deviation. Then, we convert this value to a centered percentile score to minimize the effect of right skewing—the proportion centered by doubling and subtracting 1. We multiplied cantered percentile value by the inflammatory effect score of each food parameter based on the study by shivappa et al. [10], and all foods’ DII scores were summed to obtain the overall DII score for each participant. To adjust DII for energy, we converted all dietary parameters to the amount of food intake per 1000 calories [45]. Finally, by considering energy as a denominator, 28 food parameters were used to calculate the E-DII score.

### Physical activity (PA)

The physical activity of participants in the study was evaluated using the International Physical Activity Questionnaire (IPAQ) long form. The reliability and validity of this questionnaire were previously confirmed [46]. The IPAQ-long form is a 27-item questionnaire assessing habitual PA in four domains including occupational, transportation, household/gardening, and leisure-time activities during the last 7 days. The number of days and minutes of walking, and moderate and vigorous activities per day were collected. Moreover, the metabolic equivalent (MET) minutes per week for each activity were calculated and categorized based on guidelines for data processing and analysis of the IPAQ in the high, moderate, and low PA categories [47].

### Sleep quality

Sleep quality was assessed by a previously reliable and validated Persian version of the Pittsburgh sleep quality index (PSQI) during the last mount [48]. PSQI evaluates sleep quality by 19 items in 7 components, including subjective sleep quality, sleep latency, sleep duration, sleep efficiency, sleep disturbance, use of sleep medication, and daytime dysfunction. The score for each component is in a range of 0 to 3. The sum of the components score indicates the global score (score range is 0 to 21) and scores above 5 indicate poor sleep quality [49].

### Assessment of the covariates

Demographic characteristics of participants, such as age, sex, level of education, marital status, and smoking habits were gathered by a general questionnaire in an interview. Anthropometric assessments such as weight, height, waist, hip, and waist circumference were measured based on the US National Institutes of Health protocols [50]. Moreover, to enhance the accuracy of the measurement, the assessments were in the morning while participants were fasting and emptied their bladder. Body mass index is calculated from the standard formula (BMI = weight (kg)/height2 (m)) as well as the waist-to-hip ratio (WHR = waist circumference (cm)/hip circumference (cm)).

### Statistical analysis

The characteristics of the participants were summarized using means and standard deviations (SD) for continuous variables and frequency for categorical variables. The distribution of data was evaluated using Kolmogorov–Smirnov test. An independent t-test was used for the comparison of continuous variables with a normal distribution across gender groups, while an independent Mann–Whitney U test was used for nonnormal distribution, whereas a Chi-square or Fisher’s exact test was used to compare categorical variables. Multivariate logistic regression was applied to estimate the odds ratio and 95% confidence intervals (CI) of independent and interactive association of E-DII and PA with sleep quality after stratifying participants based on gender. The interaction was evaluated by applying multiplication terms between two independent variables in the logistic model. All statistical analyses were conducted using IBM SPSS for Windows version 25.0 (IBM Corporation, Armonk, NY, USA).

## Result

A total number of 2466 participants, including 1491 women (60%) (mean (SD) age: 41.82 (6.64)) enrolled in the analysis. No significant difference was observed between gender groups for age (*p* = 0.19). The median (IQR) for E-DII was 0.8 (2.79) in men and − 0.78 (2.71) in women. Therefore, women had higher adherence to an anti-inflammatory diet compared with men in the study (*p* ≤ 0.001). Moreover, women had higher PA than men (*p* ≤ 0.001). Sleep quality was better in men (*p* ≤ 0.001). The characteristics of the participants are displayed in Table 1.

**Table 1:** Characteristics of participants

			Total *n* = 2466	Men *n* = 975	Women *n* = 1491	*P*-value
Age, mean (SD)			41.99 (6.8)	42.24 (7.03)	41.82 (6.64)	0.192
Height (cm), mean (SD)			164.16 (9.16)	172.42 (6.73)	158.76(5.95)	≤ 0.001
Wight (kg), mean (SD) Median (IQR)			72.33 (14.11) 70.80 (17.5)	80.17 (14.32) 78.7 (15.5)	67.21(11.35) 66.2 (13.6)	≤ 0.001
BMI (kg/m2), mean (SD) Median (IQR)			26.75 (4.18) 26.4 (4.96)	26.9(4.14) 26.75 (4.65)	26.66(4.21) 26.12 (5.16)	0.54
Waist circumference (cm), mean (SD) Median (IQR)			93.80 (10.34) 93.2 (12.9)	96.87(10.21) 96.3 (12)	91.8 (9.93) 91 (11.6)	≤ 0.001
Hip circumference (cm) mean (SD) Median (IQR)			102.44 (7.74) 102 (9.42)	102.71(7.65) 102.4 (9)	102.27 (7.8) 101.7 (9.7)	0.94
WHR, mean (SD)			0.92 (0.06)	0.94(0.05)	0.90(0.07)	≤ 0.001
E-DII, mean (SD) Median (IQR)			-0.10 (1.97) -0.14 (2.99)	0.72 (1.85) 0.8 (2.79)	-0.64 (1.85) -0.78 (2.71)	≤ 0.001
Marital status, n (%)		Married	1986 (80.5)	890 (91.3)	1096 (73.5)	≤ 0.001
		Single	480 (19.5)	85 (8.7)	395 (26.5)	
Education level, n (%)		Under diploma and diploma degree	239 (9.7)	155(15.9)	84 (5.6)	≤ 0.001
		Post-diploma and bachelor’s degree	654 (26.5)	263(27.0)	391(26.2)	
		Master’s and Ph.D. degree	1573 (63.8)	557(57.1)	1016(68.1)	
Smoking, n (%)		Nonsmoker	2273 (92.2)	787(80.7)	1486 (99.7)	≤ 0.001
		Ex-smoker	64 (2.6)	62 (6.4)	2 (0.1)	
		Smoker	129 (5.2)	126(12.9)	3 (0.2)	
Sleep quality, n (%)		Good	1228 (49.8)	538(55.2)	690(46.3)	≤ 0.001
		Poor	1238 (50.2)	437(44.8)	801(53.7)	
Physical activity, n (%)		low	518 (21)	186 (19.1)	332 (22.3)	≤ 0.001
		Moderate	890 (36.1)	319 (32.7)	571 (38.3)	
		High	1058 (42.9)	470 (48.2)	588 (39.4)	
Chronic diseases, n (%)		Yes	408 (16.5)	138 (14.2)	270 (18.1)	0.01
		No	2058 (83.5)	837 (85.8)	121 (81.9)	
Chronic diseases, n (%)		Hypertension	173 (7.0)	81 (8.3)	92 (6.2)	0.042
		Diabetes	78 (3.2)	35 (3.6)	43 (2.9)	0.328
		Cancer	19 (0.8)	3 (0.3)	16 (1.1)	0.34
		Depression	188 (7.6)	38 (3.9)	150 (10.1)	≤ 0.001

Values are means (SD) for continuous variables with normal distribution; median [IQR] report for continuous variables with non-normal distribution and count (percentage) for categorical variables. Independent sample t-test and independent Mann–Whitney U test were used for continuous variables, and Chi-square or Fisher’s exact test was used to compare categorical variables. SD: standard deviation, IQR: interquartile range, n: number, BMI: body mass index, WHR: waist-to-hip ratio

Table 2 shows the association between gender, energy-adjusted dietary inflammatory index (E-DII), and physical activity (PA) with poor sleep quality. Women had 70% increased odds for poor sleep quality (OR: 1.7, 95% CI: 1.39_2.09) compared with men. No significant association was observed between E-DII and sleep quality (OR: 0.96, 95% CI: 0.92_1.01). Also, there was no significant association between the levels of PA and sleep quality (OR: 1.09, 95% CI: 0.9_1.31). No interactive association was observed on the association between E-DII and PA levels with sleep quality.

**Table 2:** Association between gender, energy-adjusted dietary inflammatory index (E-DII), and physical activity (PA) with poor sleep quality

Variable			Unadjusted Odds ratio (95% CI)	*P* value	Adjusted^1^ Odds ratio (95% CI)	*P* value
Gender	Men		1	1	1	1
	Women		1.43 (1.22_1.68)	≤ 0.001	1.7 (1.39_2.09)	≤ 0.001
E-DII			1 (0.96_1.04)	0.98	0.96 (0.92_1.01)	0.08
PA	Low		1	1	1	1
	Moderate		0.91 (0.74_1.12)	0.37	0.92 (0.74_1.15)	0.49
	High		1.09 (0.91_1.3)	0.34	1.09 (0.9_1.31)	0.39
E-DII*PA	Low PA		1	1	1	1
	Moderate PA		1.03 (0.94_1.13)	0.49	1 (0.91_1.1)	0.98
	High PA		1.01 (0.94_1.08)	0.79	0.97 (0.91_1.04)	0.42

1 Adjusted for age, body mass index (BMI), waist-to-hip ratio (WHR), energy intake, educational level, marital status, smoking, chronic disease (hypertension, diabetes, cancer, depression)

## Discussion

This study observed no significant association between E-DII, PA, and sleep quality. Gender differences were found in the study and women had higher odds of poor sleep quality. There was no significant interactive association between E-DII and PA with sleep quality.

The present study found no significant association between E-DII and poor sleep quality. Previously, a study by Godos et al. on 1936 South Italian adults indicated a protective effect for higher adherence to an anti-inflammatory diet and better sleep quality but the study did not evaluate gender differences [13]. A study on 379 college students in the United Arab Emirates (UAE) found no significant association between E-DII and sleep quality [12] meanwhile, another study on 249 female students in Iran reported a significant direct association between E-DII and poor sleep quality [11]. Also, Setayesh et al. in a study on 219 obese and overweight women report that higher DII scores is associated with poor sleep quality [51]. Various differences between the mentioned studies and the current study may explain the conflict in findings such as mean age of participants (college students and adults), gender (two studies were only on women), studies sample size, and sociodemographic variables like smoking and alcohol consumption assessments in study might also explain the current conflicts.

We found no significant association between PA levels and sleep quality. Several previous studies also reported no significant association between PA and sleep quality [52, 53], whereas some others found a moderately significant association [25, 54]. This can be due to the different methods for evaluating PA. There are numerous ways to assess PA, in our study, we used the IPAQ questionnaire and categorized the overall PA into 3 levels but it’s also available to assess the PA in different methods such as PA domains (occupational, domestic, transportation, and leisure time) as well as using total METs of walking, moderate and vigorous PA; considering PA intensity; time and type of PA and using device-based measurements to evaluate PA and sleep. So, these differences may be enrolled in the association between PA and sleep [55, 56]. In this study, there was no significant interactive association between E-DII and PA with sleep quality. Although E-DII and PA activity level was not significantly associated with sleep quality, to differentiate appropriate non-pharmacological interventions for improvement of sleep quality different aspects of diet and PA should be evaluated in further investigations.

Women had higher odds of poor sleep quality in comparison with men which is consistent with some previous studies [37–39]. The impression of gender on sleep has been previously under discussion due to differences in rates of sleep disorders between genders [43, 57–59]. The diversity between sleep-related outcomes prevalence in men and women may come from several relevant differences. Differences in biological hormones, psychosocial characteristics, stress, and genetic and gut microbiota composition have been hypothesized to influence sleep. It’s suggested that women’s neurobiological mechanisms and hormones, especially fluctuation of ovarian hormones, may affect sleep because of an interaction between hormone levels and circadian rhythm, however, there is a need for future investigations to clarify the underlying mechanism for this association [43, 60]. Higher stress levels may lead to poor sleep quality [8, 61–63]; while there is a probable gender differences in stress response [64, 65]; so, it can be presumed that the impact of gender on stress response may have a role in the association between gender and sleep quality. Moreover, the interplay among sex differences, gut microbiota, and sleep should be considered in this subject [41, 66]. Sex-related features may affect gut microbiota diversity which can impact the gut-brain axis (GBA) [67]. Alteration in inflammatory markers such as IL-6, psychological factors such as stress and depression, or circadian rhythm can be the consequences of these differences that may lead to disruption in sleep [32, 67–69]. However, although one of the roles of diet and PA in health outcomes comes from their role on gut microbiota, the effect of probable sex differences in gut microbiota composition and its impact on sleep and different health conditions is not yet well demonstrated and there is need for further investigations.

The strengths of the study are the adjustments for a wide range of potential confounders and the evaluation of the independent and interactive association between E-DII, PA, and sleep quality. The limitation of our study is the cross-sectional design. Moreover, the questionnaires used for measuring dietary intake (FFQ), PA (IPAQ), and sleep quality (PSQI) may have recall bias. Using different methods for the assessment of variables in further studies may lead to different findings. For instance, assessment of dietary status using dietary records, or measuring levels of inflammatory cytokine for evaluation of dietary and inflammatory status; using sleep assessment devices such as actigraphy or polysomnography; and PA assessment devices such as accelerometer. The result should not be generalized to the general population because the study was conducted on medical university employees; therefore, participants’ educational level, health literacy, and access to medical services and information differ from the general population. There is a need for prospective studies and interventional trials to investigate independent and interactive associations between dietary patterns, PA, and sleep quality.

## Conclusions

In conclusion, the study indicates a gender difference in sleep quality. No association was observed between E-DII and PA with sleep quality. It’s suggested that future studies use different methods for evaluating PA and sleep, such as devise-based methods to investigate the independent and interactive association of diet and PA with sleep quality. Future prospective studies are required to confirm these findings.

## Data Availability

The datasets analyzed in the current study are available from the corresponding author upon reasonable request.

## Abbreviations

BMI: Body mass index
CI: Confidence interval
CRP: C-reactive protein
CVD: Cardiovascular diseases
E-DII: Energy-adjusted dietary inflammatory index
FFQ: Food frequency questionnaire
GBA: Gut-brain axis
IL-6: Interleukin-6
IPAQ: International Physical Activity questionnaire
IQR: Interquartile range
OR: Odds ratio
PA: Physical activity
PSQI: Pittsburgh sleep quality index
SD: Standard deviation
SUMS EHCS: Shiraz University of Medical Sciences Employee Health Cohort Study
TNFα: Tumor necrosis factorα
WHR: Waist-to-hip ratio
